# Inner Surface Morphology and Roughness Evolution of Pilgering Thick-Walled Tubes

**DOI:** 10.3390/ma16247618

**Published:** 2023-12-12

**Authors:** Ran Li, Pengfei Jin, Weijie Wang, Cheng Zhang, Xingwu Du, Jinfeng Huang

**Affiliations:** 1 State Key Laboratory for Advanced Metals and Materials, University of Science and Technology Beijing, Beijing 100083, China; iraniggia@sina.com (R.L.);; 2 Changzhou Xingtong Machinery Manufacturing Co., Ltd., Changzhou 213100, China

**Keywords:** thick-walled seamless tube, Pilger rolling, inner wall roughness, inner surface topography

## Abstract

A hot-working die steel thick-walled tube Pilger rolling test was carried out using an LG40 Pilger mill, and the morphology and roughness evolution of the inner surface were examined using a white-light interferometer. The experimental results showed that micro-wrinkles formed on the basis of the original inner surface morphology, the altitude difference (*Sz*) between the peaks and valleys of the inner surface profile increased from 3.18 to 3.686 μm, and *Sa* increased from 0.722 to 0.892 μm in the diameter reduction zone. As the tube continued to feed into the wall thickness reduction zone, the micro-wrinkles gradually flattened, *Sz* and *Sa* were decreased to 1.625 and 0.174 respectively, and *Sa* maintained a slight fluctuation of 0.174~0.2 μm in the final sizing zone. From the diameters of the roller groove and taper of the mandrel, the three-dimensional strain of the tube in the wall thickness reduction zone was calculated, and the strain state of the tube in the complete deformation zone could be analyzed by finite element simulations. We found that in the diameter reduction zone, the inner surface was not supported by the mandrel and was free, while micro-wrinkles formed under circumferential compressive strain. In the wall thickness reduction zone, the deformation of the inner surface was controlled by the mandrel, and the micro-wrinkles were gradually flattened by radial compressive strain. The ratio of radial to circumferential strain was the key to flattening the micro-wrinkles, and when the ratio increased, the inner surface roughness (*Sa*) was reduced to 0.174 μm. In the sizing zone, the radial and circumferential strains were small, and the inner surface roughness showed no obvious fluctuation.

## 1. Introduction

Small-caliber thick-walled tubes serve as key components with important roles in fluid transmission in precision equipment for hydraulic systems. The inner surface of the tube is subjected to repeated impacts from the hydraulic oil inside, as well as internal high pressure and alternating cycle stress. The fatigue life of tubes subjected to internal high pressure appears to be greatly influenced by the changes in surface topography, which include surface defects and surface roughness. Surface roughness is the unevenness of a workpiece surface with small spacing and tiny peaks and valleys [1,2,3]. Dicecco et al. [4] reported that a higher surface roughness corresponded to worse fatigue failure resistance. High surface roughness may be the origin of early fatigue cracks [5,6]. Therefore, reducing surface roughness is mainly used to improve fatigue life. Currently, small-diameter thick-walled tubes (outer diameter/wall thickness ≥ 3) for hydraulic machinery with inner surface roughness *Sa* ≤ 0.4 μm are mainly prepared by deep-hole drilling and multiple honing passes, which have the disadvantages of low efficiency and large resource consumption. Pilger cold rolling has been widely used in the preparation of high-precision thin-walled seamless tubes due to its high efficiency, low consumption, high dimensional accuracy, and good surface quality of the rolled seamless tubes [7,8,9]. If Pilger methods are used to prepare small-caliber thick-walled tubes, they can replace multiple honing and improve efficiency. However, due to the complexity of the Pilger cold rolling forming process and groove geometry, the test cost remains high, with long cycling times [10]. As a result, few studies have investigated the preparation of small-caliber thick-walled seamless tubes by Pilger cold rolling.

**Figure 1:**
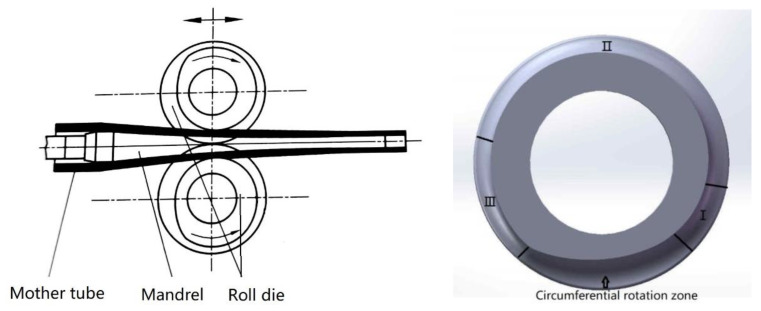
Cold pilgering process and roller groove.

The Pilger rolling process is shown in Figure 1. In this, two rollers are typically distributed up and down, and the circumferential surface of the rollers contains grooves that define the deformation path of the tube. The mandrel remains fixed inside the tube without axial displacement. The process of the rollers moving from the back limit position to the front limit position is known as the positive stroke, while the reverse is known as the reverse stroke. Thus, the rollers can cycle back and forth to realize the periodic rolling deformation of the mother tube in the area surrounded by the two rollers and a mandrel [11]. The deformation of the tubes is controlled by the roller grooves, with the groove consisting of a rotating zone and working zone (including the diameter reducing zone—I, wall thickness reducing zone—II, and sizing zone—III). The tube in the rotating zone will not undergo deformation, mainly to complete rotation along the circumference and axial feeding of the tube. The mother tube will be reduced in diameter and wall deformation in I and II, and the precision of the final inner and outer diameter size will be controlled in III [12].

At present, various studies have reported on the surface quality in cold pilgering. Abe H et al. [13] proposed that the critical reduction in height upon crack initiation *φ_c_* was a good measure of material deformability in pilgering, with *φ_c_* the logarithm of the height ratio before and after compression. The selection of an appropriate *φ_c_* could assist in preventing the formation of cracks during the cold rolling process. Abe H et al. [14] studied inner fissure formation, and the factors that affected, it in Zr alloy tubes. The compressive properties of the tube, which were related to the heat treatment conditions, affected inner fissure formation during cold pilgering. Zhang et al. [15] simulated the pilgering process of zirconium alloy tubes rolled with different roller grooves using finite element simulations, and analyzed the influence of groove parameters on the rolling force and surface cracks of the finished tubes during the cold pilgering forming process. Montmitonnet P et al. [16] studied the pilgering process of Zircaloy-4 alloy tubes and proposed that the normal stress at the bottom of the rolling groove affected the surface morphology of the tube. The study established a plane model to calculate the normal stress and rolling force at the bottom of the roller grooves; however, certain differences were observed between the results and the actual rolling force. Abe H et al. [17] proposed a method to calculate the thickness of the oil film on the Pilger rolled tube surface using the Reynolds equation. The study indicated that the stroke speed and feed rate had significant effects on the oil film thickness. However, the rolling test results showed that the inner surface roughness of the steel tube after pilgering was greater than before.

In this study, a Pilger rolling test was carried out on a thick wall tube, and the surface morphology and inner surface roughness of the thick wall tube were obtained. ABAQUS software (version 2022) was used to simulate the strain state of the inner surface of the thick-walled tube during pilgering, and, according to the angles of the rolled groove and the mandrel, the variation of the simulation results was verified by formula calculations. The variation rule of the inner surface roughness and mechanism of morphology evolution were analyzed according to the strain state of the inner surface.

## 2. Experiment and Simulation

### 2.1. Experimental Materials

The test material consisted of hot-working die steel; the microstructure was predominantly tempered martensite. Table 1 presents the chemical composition of the experimental materials.

### 2.2. Pilgering Experiment

**Figure 2:**
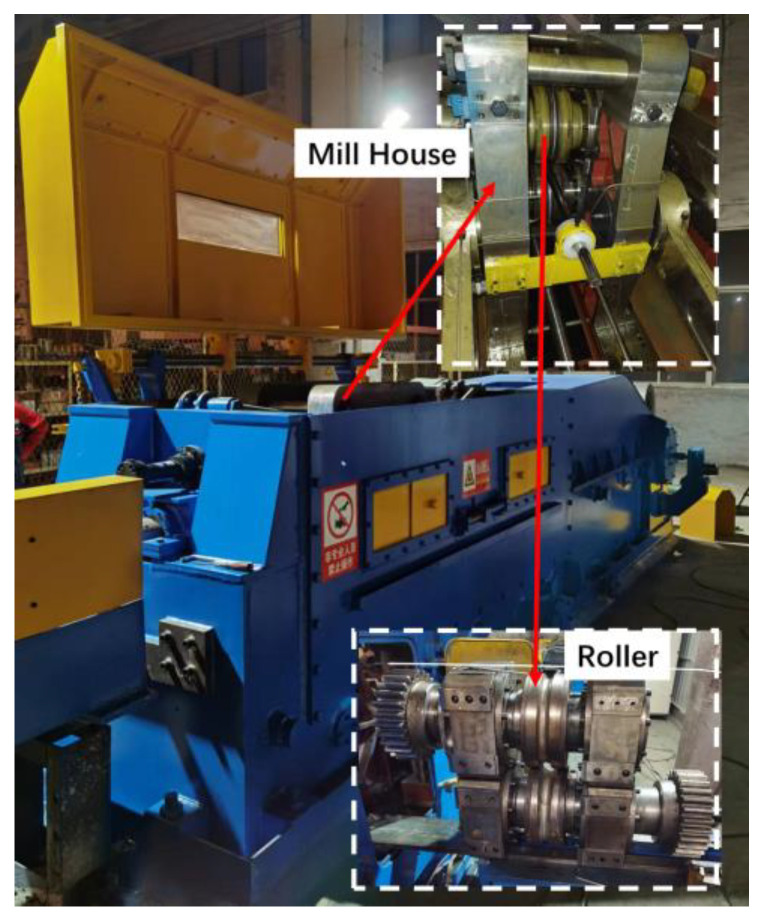
LG40 Pilger mill.

**Figure 3:**
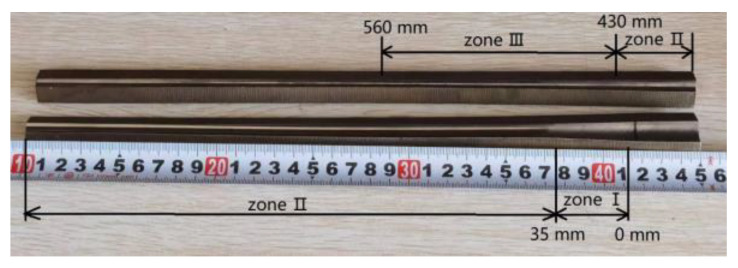
The deformation cone of the tube.

**Figure 4:**
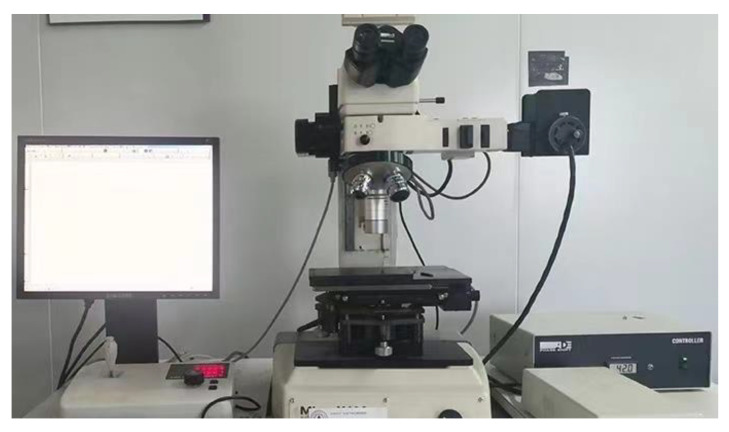
White-light interferometry—MicroXAM-3D.

In the cold pilgering test, the mother tube had an outer diameter (OD) of 47 mm, 14.5 mm wall thickness (WT), and length of 1000 mm; the finished tubes were 33 mm in OD and 11 mm in WT. The inner surface roughness (*Sa*) of the mother tube was 0.772 μm, which was obtained by honing. An LG40 test Pilger mill (Yuyao Xingtong Machinery Manufacturing Factory, Yuyao, China) was used, as shown in Figure 2. The pilgering parameters were as follows: feed rate of 3 mm/stroke, turn angle of 53°/stroke, and stroke speed of 60 strokes/min. Rolling was stopped after the tube was fed in 500 mm. The selected cone contained the complete deformation zone, which included a diameter reduction zone (I, 0–30 mm), a wall thickness reduction zone (II, 40–430 mm), and a sizing zone (III, 430–560 mm), and was cut along the axial direction. As shown in Figure 3, to facilitate the observation of the inner surface morphology, the cone was divided into two sections. The inner surface morphology and roughness of the tubes were measured by white-light interferometry (MicroXAM-3D, KLA Instruments, Milpitas, CA, USA), as shown in Figure 4, to quantitatively and qualitatively describe roughening, and analyzed using Gwyddion software (version 2.61). Measurement information is shown in Table 2.

In this study, the inner surface roughness of the tube was characterized by determining a number of statistical parameters, as follows [18]:

*Sa*: arithmetic average of the absolute values of height differences at each point within a defined area (*A*), as defined in Equation (1)

(1) $$Sa=\frac{1}{A}\iint_{A}^{\;}|Z\left(x,y\right)|dxdy$$

*Sq*: root mean square value of the ordinate values within a defined area (*A*), as defined in Equation (2)

(2) $$Sq=\sqrt{\frac{1}{A}\iint_{A}^{\;}Z^{2}\left(x,y\right)dxdy}$$

*Sz*: sum of the maximum peak height value (*Sp*) and the maximum pit height value (*Sv*) within a defined area, as defined in Equation (3)

(3) Sz=Sp+Sv

### 2.3. Simulation of Pilgering

**Figure 5:**
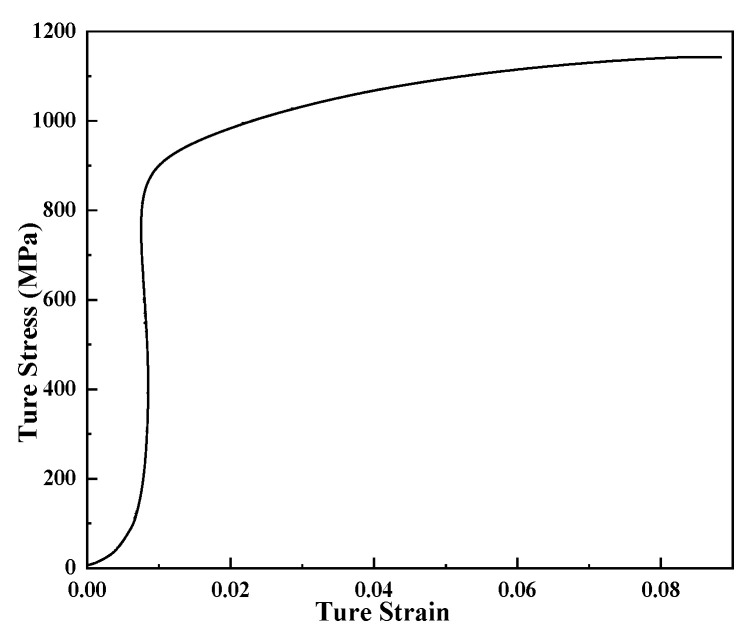
True strain–stress curve of hot-working die steel at room temperature.

The true stress–true strain curve of the material was obtained by tensile testing at room temperature, as shown in Figure 5. The elastic modulus of the material was determined as 200 GPa and the Poisson’s ratio was 0.3. These material parameters were imported into ABAQUS finite element software as the material attributes of the tube model for analysis.

**Figure 6:**
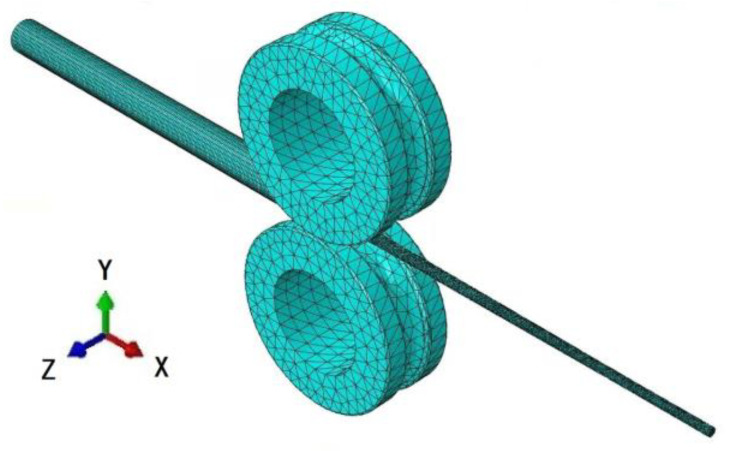
Schematic diagram of the finite element model.

The finite element model of pilgering is shown in Figure 6. Friction between the roller, tube, and mandrel was generated during the pilgering process. In the finite element model, the type of friction was defined as Coulomb friction, and the friction coefficient between the different contact surfaces was set to 0.1 [19]. The phenomena that occurred during pilgering introduced a strong non-linearity to the model, and these primarily connected with the cyclical nature of the process and complex contact problems [20]. To simplify the computing tasks, the following additional assumptions were made: The rollers and mandrel were considered incompressible rigid bodies, and in the pilgering process, the inertial forces were not considered. The model did not take into account the effects of temperature and strain rate. In addition, the feed rate was 3 mm/stroke, the turn angle was 53°, and stroke speed was 60 strokes/min.

## 3. Results and Validation

### 3.1. Morphology and Roughness of the Inner Surface

**Figure 7:**
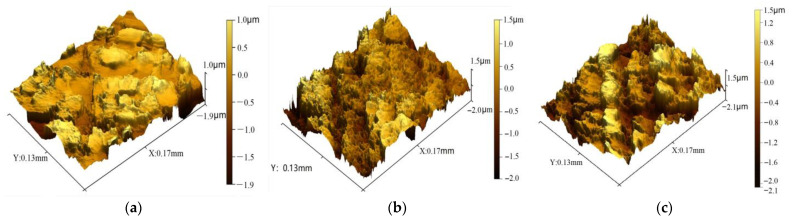
Inner surface morphology of zone I (**a**): 10 mm; (**b**): 20 mm; (**c**): 30 mm.

In zone I, the shape of peaks and valleys on the inner surface of the mother tube consisted of tiny platforms, as shown in Figure 7a. With the progression of the Pilger process, the diameter decreased and the inner surface shrank, while the peaks and valleys became denser and sharper. The peak bulges increased and the valleys became further depressed. The altitude difference between the peaks and valleys (*Sz*) increased from 2.9 to 3.686 μm within the range of 10–30 mm. *Sa* has been used to describe how the surface heights fluctuated around the mean plane. The average absolute values of the altitude differences of each point on the observation surface (*Sa*) increased from 0.722 to 0.892 μm. The morphology of the inner surface became obviously toughener, as shown in Figure 7.

**Figure 8:**
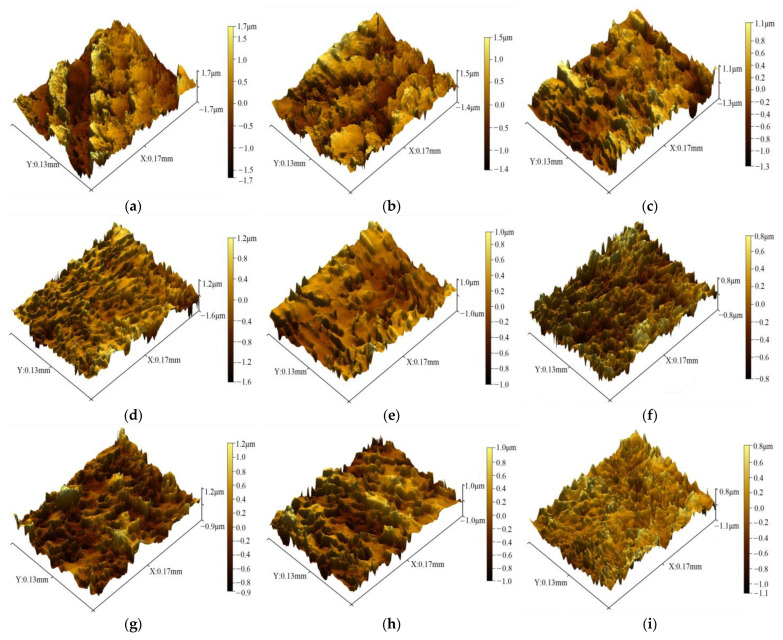
Inner surface roughness of zones II and III: (**a**): 40 mm, (**b**): 60 mm, (**c**): 120 mm, (**d**): 200 mm, (**e**): 280 mm, (**f**): 320 mm, (**g**): 390 mm, (**h**): 430 mm, and (**i**): 470 mm.

In zone II, as the Pilger process proceeded, the diameter and wall thickness of the tube simultaneously decreased. As shown in Figure 8a–h, the area was occupied by the bottom of the peaks and top of the valleys on the inner surface, which decreased gradually. The height of the peaks and the depth of valleys were significantly lower than in zone I, and the peaks were inclined, which was related to the simultaneous axial feeding and circumferential rotation of the tube during the Pilger rolling process. *Sz* decreased from 3.686 to 1.625 μm, and *Sa* decreased from 0.892 to 0.174 μm. Although *Sz* and *Sa* fluctuated slightly, the overall trend was downward. In zone III, *Sa* was stable at 0.177 μm, as shown in Figure 8i.

**Figure 9:**
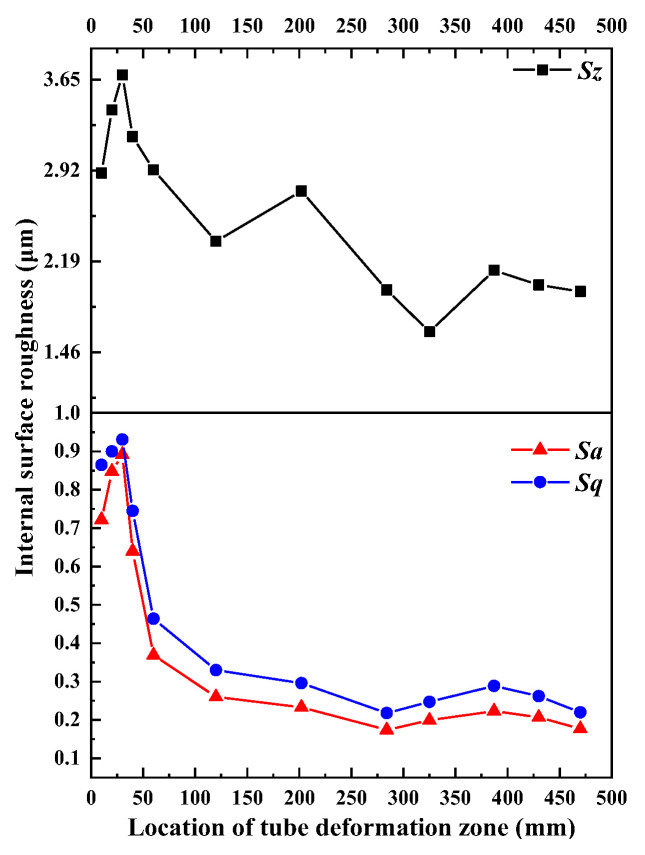
Evolution of inner surface roughness in the working zone.

The surface roughness parameters (*Sz*, *Sa*, and *Sq*) were extracted to quantitatively describe the change in roughening defects. The evolution of inner surface roughness during pilgering is shown in Figure 9, with *Sq* equivalent to the square root of mean squares of the observed surface. This was the quadratic average of the asperities and was possibly applicable for identifying the significant variations in surface characteristics. Figure 7 shows the values of *Sz* > *Sq* > *Sa* in the entire working zone, with *Sz*, *Sa*, and *Sq* following the same trend, which increased in zone I, decreased and stabilized in zone II, and finally was not obviously changed in zone III.

### 3.2. Simulation Results

**Figure 10:**
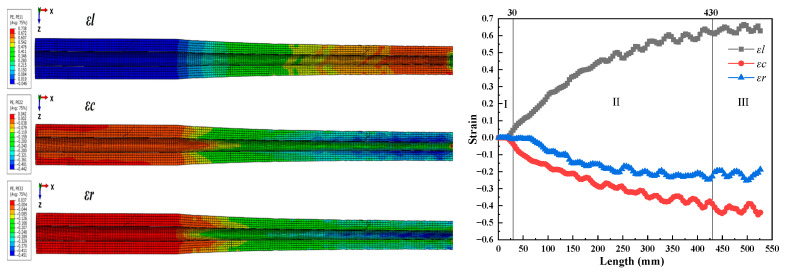
The numerical simulation results for the entire deformation cone.

Figure 10 shows the numerical simulation results for the entire deformation cone, indicating that relative deformation was very small in three directions between the two adjacent sections. This reflected the processing characteristics of multi-pass small-deformation formation of the Pilger rolled tube. As shown in Figure 8b, the circumferential strain on the inner surface consisted of compressive strain, and the absolute value of strain increased from 0.082 to 0.452. Initially in zone II, no significant changes were observed in radial strain, which remained around zero, but as pilgering continued, the strain value increased from 0.00426 to 0.25175. Because the mother tube was not in contact with the mandrel in zone I, the wall thickness increased slightly under the radial and circumferential stress. This was initially compressed to the wall thickness of the mother tube, which gradually decreased in zone II. The axial strain was the tensile strain, which increased from 0 to 0.6645, and the amplitude was significantly higher than the axial and radial strains.

**Figure 11:**
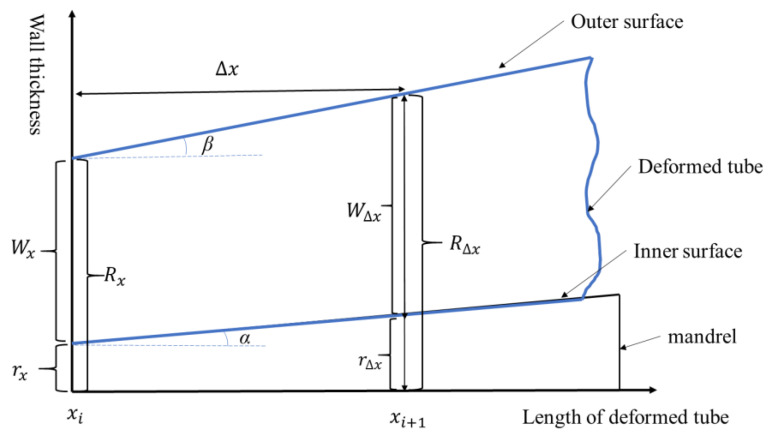
The axial cross-section of the tiny area of the deformation cone at zone II.

The deformed tube between the front and rear limit positions during the Pilger rolling process was labeled the as deformation cone. The outer surface contour of the deformation cone (angle between the bottom of the roller groove that was spread out along the circumferential direction and the horizontal line) and the inner surface contour (the taper curve of the mandrel surface) consisted of two smooth curves. After each pilgering stroke, the tube was fed a certain distance in the front limit direction. The contours of the inner and outer surfaces of the tube fed in a single stroke were considered approximately straight lines. The angles between the inner and outer surface contours and the horizontal line were α and β respectively, as shown in Figure 11.

To determine the deformation amount of any section (such as *x_i_*) under each stroke, we had to determine the axial distance Δ*x* of the section after a stroke, namely the *x_i+_*_1_ section:

(4) $$R_{∆x}=R_{x}+{∆}_{x}tan\beta =r_{∆x}+W_{x}+{∆}_{x}\left(tan\beta -tan\alpha \right),$$

(5) $$r_{∆x}=r_{x}+{∆}_{x}tan\alpha ,$$

(6) $$V_{m}=\pi m\left(R_{m}^{2}-r_{m}^{2}\right)=\pi \int_{0}^{{∆}_{x}}\left(R_{m}^{2}-r_{m}^{2}\right)d\left({∆}_{x}\right),$$

(7) $$∆x=\frac{\sqrt{W_{x}^{2}{\left(2r_{x}+W_{x}\right)}^{2}+4\frac{V_{m}}{\pi }\left[r_{x}\left(tan\gamma -tan\alpha \right)+W_{x}tan\gamma \right]}-W_{x}\left(2r_{x}+W_{x}\right)}{2\left[r_{x}\left(tan\gamma -tan\alpha \right)+W_{x}tan\gamma \right]},$$

(8) $$\varepsilon_{l}^{i}=\frac{R_{0}^{2}-r_{0}^{2}}{R_{i}^{2}-r_{i}^{2}}-1,$$

(9) $$\varepsilon_{r}^{i}=\frac{W_{i}-W_{m}}{W_{m}},$$

(10) $$\varepsilon_{c}^{i}=\frac{r_{i}-r_{m}}{r_{m}},$$

where $R_{x}$ and $R_{∆x}$ denote the outer radius of the deformed cone before and after rolling per stroke; respectively,$r_{x}$ and $r_{∆x}$ signify the inner radius of the deformed cone before and after rolling per stroke, respectively;$W_{x}$ and $W_{∆x}$ are the wall thickness of the deformed cone before and after rolling with per stroke, respectively;$R_{m}$, $r_{m}$, and $W_{m}$ signify the outer radius, inner radius, and wall thickness of the mother tube, respectively; β is the angle between the bottom of the roller groove spread out along the circumferential direction and the horizontal line; α is the taper of the mandrel; and $\varepsilon_{l}^{i}$, $\varepsilon_{r}^{i}$, and $\varepsilon_{c}^{i}$ indicate the axial, radial, and circumferential strain variables of section *i*, respectively.

**Figure 12:**
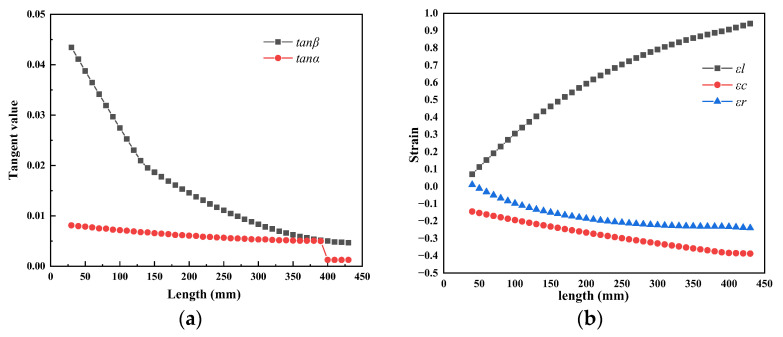
(**a**) The tangent value of the angles; (**b**) strain of the deformation cone in zone II.

The tangent angles of the roller groove and mandrel cone are shown in Figure 12a, and the results of calculation are detailed in Table A1 in Appendix A. The outer contour of the mandrel consisted of a curve with decreasing taper, and gradually transitioned to a horizontal line at the end of zone II. We chose the size of the finished tube as the starting point of the calculation (i.e., *r_x_* = 5.5, *R_x_* = 16.5 mm), and the starting point of the wall thickness reduction zone was the end point of calculation. The theoretical strain of each section in the deformation cone in zone II is shown in Figure 12b.

As shown in Figure 12b, the radial strain of the inner surface was compressive strain, which increased from 0.010 to 0.24 with pilgering; however, the increment gradually decreased. This could be attributed to the outer contour of the mandrel consisting of a curve with very small tapers, and the tangent value of the mandrel in zone II ranged from 0.0081 to 0.005. The circumferential strain value on the inner surface was negative, indicating compressive strain, and the value demonstrated an increasing trend from 0.1446 to 0.388. The axial strain value of the inner surface was positive, indicating tensile strain, and the value demonstrated an increasing trend from 0.078 to 0.9407. The value of the axial strain was significantly higher than for the other strains. The increment strains in the three directions of the thick-wall surface were very small during the cold pilgering process, which indicated the processing characteristics of multi-pass small-deformation formation of the periodic rolled tube.

## 4. Discussion

**Figure 13:**
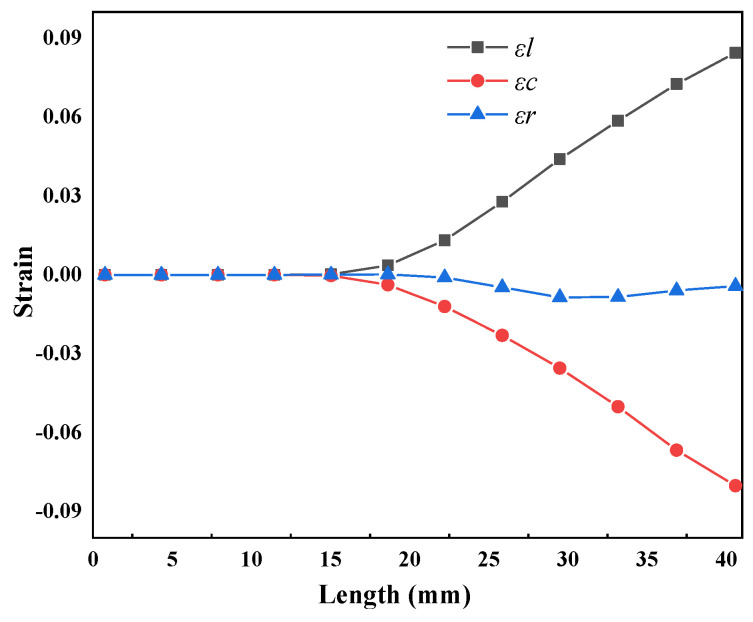
The simulation results of strain in the deformation cone of zone I.

During the pilgering process, the inner and outer diameters of the mother tube decreased and the wall thickness slightly increased in zone I. The inner surface was free without contacting the mandrel and was not restricted by the mandrel [13]. As shown in Figure 13, the axial tensile strain and circumferential compressive strain were generated on the inner surface of the deformation cone under the action of the roller groove in zone I. The original peaks and valleys on the inner surface of the mother tube squeezed each other, forming micro-wrinkles on the original surface morphology under circumferential compressive stress, and the altitude difference between the peaks and valleys increased. The new peaks and valleys were distributed along the rolling direction under axial tensile stress, and micro-wrinkles formed on the original surface, where *Sz* increased from 3.18 to 3.686 μm, and the average roughness (*Sa*) increased from 0.722 to 0.892 μm. At this stage, both the grain size and inherent slip systems of the material affected the surface roughness [13]. The surface roughness deteriorated, and the influence of circumferential plastic strain showed a significant effect of surface roughening in zone I. This result is in agreement with Abe H et al. [13,14]; that is, the free surface is subjected to circumferential strain and micro-wrinkles are formed, and with the increase in circumferential strain, the micro-wrinkles depth increases. Under the high circumferential compressive strain, micro-wrinkles may develop into cracks.

**Figure 14:**
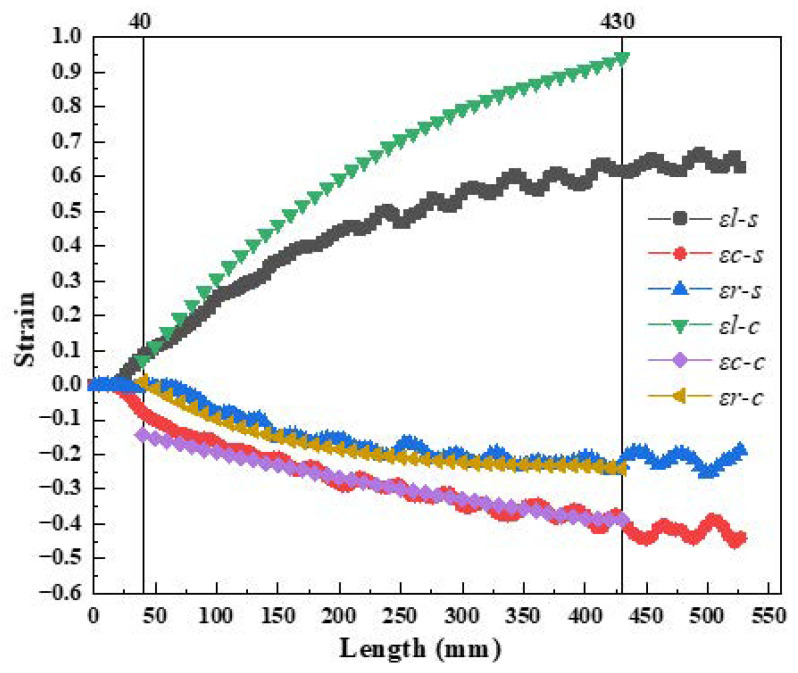
Comparison of simulated and calculated strain.

Figure 14 shows a comparison of the simulated and calculated strain. We observed that the simulated value of radial strain was slightly lower than calculated in the initial stage of zone II, primarily because the formula did not account for the increase in wall thickness in zone I. The calculated value of axial strain was greater than the simulated value, primarily because the calculated results consisted of the overall strain of the cross-section, while the strain of the simulation was on the inner surface. However, the deformation of the thick-walled tubes on the inner and outer surfaces was uneven during the rolling process, and deformation on the outer surface was greater than the inner surface. As a result, the calculated results were greater than the simulation results. From the perspective of the entire deformation zone, the strain curve trends of the simulation results and calculation in the three directions were consistent in zone II. Therefore, the calculation results had a positive effect on the verification of the simulation results.

**Figure 15:**
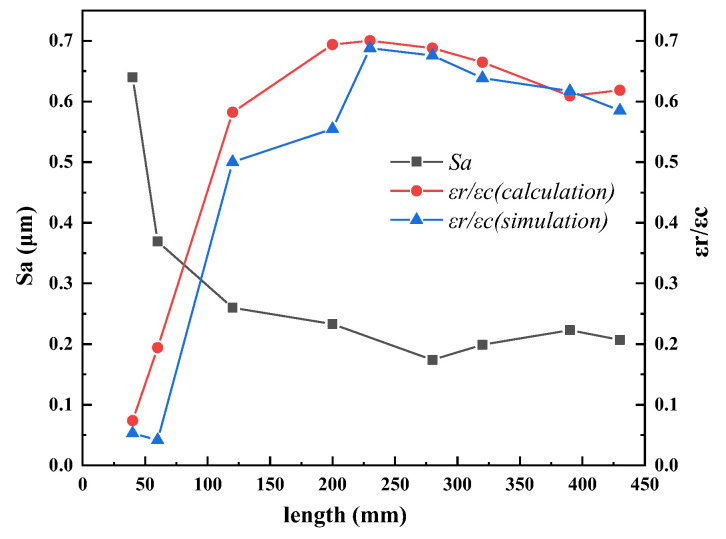
The relationship of (*ε_r_/ε_c_*) and *Sa* in zone II.

The plastic deformation state affected the formation of inner micro-wrinkles during pilgering. The micro-wrinkles on the inner surface of the tube formed by circumferential strain in zone I, while in zone II, deformation of the inner surface was in contact with the mandrel and restricted by the mandrel. The variation in inner surface roughness could not be explained by only the circumferential strain in zone II, and the radial stress acted on the inner surface to flatten the micro-wrinkles. Figure 15 shows the relationship between calculated and simulated (*ε_r_/ε_c_*) and *Sa* in zone II, indicating that the variation in (*ε_r_/ε_c_*) tended to be consistent with the simulation and calculations. As (*ε_r_/ε_c_*) increased, the inner wall surface roughness decreased. In the range of 40–230 mm, the increment of *ε_r_* was larger than *ε_c_*, and the (*ε_r_/ε_c_*) increased from 0.074 to the maximum value of 0.7. In this stage, *ε_r_* played a major role in flattening, and *Sa* decreased from 0.64 to 0.26 μm. In the range of 230–430 mm, *ε_r_/ε_c_* was stable within the range of 0.618–0.7, while the corresponding *Sa* fluctuated within the range of 0.174–0.233 μm.

As the sizing zone, the function of zone III was to make the inner and outer diameter of the tube more circular. At this time, the inner and outer diameters of the tube have basically reached the final size, and the variations in circumferential and radial strain were very small, with little influence on the surface roughness, and *Sa* was stable at 0.177 μm. Therefore, the ratio of radial and circumferential strain in zone II had a significant effect on the surface roughness of the tube during the Pilger rolling process of thick-walled tubes. These results may allow manufacturers to choose appropriate cold pilgering conditions for thick-walled tubes.

## 5. Conclusions

In this study, we analyzed the evolution of inner surface morphology and roughness of thick-walled tubes during the Pilger cold rolling process. The strain state of the inner surface was obtained through theoretical calculation and finite element simulations, and the key influencing factors on the inner surface morphology during the Pilger cold rolling process were determined. The following conclusions were drawn:

(1) In the diameter reduction zone, the original peaks and valleys of the inner surface became squeezed by each other, forming micro-wrinkles under circumferential compressive stress, and the new peaks and valleys were distributed along the rolling direction under axial tensile stress. In the wall thickness reduction zone, the tops of the peaks and bottoms of valleys became sharper, and radial stress acted on the inner surface to flatten the micro-wrinkles, which remained in the sizing zone.
(2) During the Pilger process, the variation in the inner surface roughness (*Sa*, *Sz*, and *Sq*) of the tube tended to remain the same, increasing the diameter reduction zone, decreasing the wall thickness reduction zone, and stabilizing the sizing zone.
(3) The increment strains in the three directions of the thick-walled surface were very small during the cold pilgering process, which reflected the processing characteristics of multi-pass small-deformation formation in the periodic rolled tube.
(4) In the wall thickness reduction zone, (*ε_r_/ε_c_*) was negatively correlated with the inner surface roughness, and as (*ε_r_/ε_c_*) increased, the inner surface roughness decreased.

## Figures and Tables

**Table 1:** Chemical composition of the experimental materials (wt/%).

C	Si	Mn	*p*	S	Ni + Cr + Mo	W	V	Fe
0.29	0.19	0.14	≤0.015	≤0.015	5.0	0.37	0.63	Bal

**Table 2:** Measurement Info of MicroXAM-3D.

Range of the vertical scan	30 μm
Vertical scanning resolution	<0.1 nm
View field	0.17 mm × 0.13 mm
Resolution	752 × 480 pixels

## Data Availability

Data are contained within the article.

## Appendix A

**Table A1:** The results of formula calculations for the strain and OD/ID.

Length/mm	tanβ	tanα	*Rx*/mm	*rx*/mm	*ε_l_*	*ε_c_*	*ε_r_*
40	0.0411	0.00795	22.2135	7.6725	0.070785	−0.14464	0.010218
50	0.03875	0.00785	21.826	7.594	0.111446	−0.1533	−0.01133
60	0.03645	0.0077	21.4615	7.517	0.151797	−0.1618	−0.03142
70	0.03415	0.0075	21.12	7.442	0.191614	−0.17011	−0.05008
80	0.0319	0.00745	20.801	7.3675	0.230624	−0.17831	−0.06729
90	0.02965	0.00725	20.5045	7.295	0.268578	−0.18636	−0.08309
100	0.02745	0.00715	20.23	7.2235	0.305217	−0.19426	−0.09749
110	0.02525	0.00705	19.9775	7.153	0.340245	−0.20206	−0.11048
120	0.02305	0.0069	19.747	7.084	0.373371	−0.20971	−0.12208
130	0.02095	0.00675	19.5375	7.0165	0.404395	−0.21721	−0.13234
140	0.0195	0.0067	19.3425	6.9495	0.43368	−0.22462	−0.1415
150	0.01865	0.00655	19.156	6.884	0.462061	−0.23191	−0.14998
160	0.01775	0.00645	18.9785	6.8195	0.489825	−0.23906	−0.15795
170	0.0169	0.00635	18.8095	6.756	0.516874	−0.24611	−0.1654
180	0.0161	0.0062	18.6485	6.694	0.543225	−0.25302	−0.17238
190	0.0153	0.00615	18.4955	6.6325	0.568813	−0.25983	−0.17888
200	0.01455	0.00605	18.35	6.572	0.593589	−0.26655	−0.18491
210	0.01375	0.006	18.2125	6.512	0.617465	−0.2732	−0.19047
220	0.0131	0.00585	18.0815	6.4535	0.640483	−0.27973	−0.1956
230	0.01235	0.0058	17.958	6.3955	0.662617	−0.28616	−0.20033
240	0.0117	0.0057	17.841	6.3385	0.683777	−0.29251	−0.20463
250	0.0111	0.00565	17.73	6.282	0.704056	−0.29878	−0.20856
260	0.01045	0.00555	17.6255	6.2265	0.723396	−0.30497	−0.21211
270	0.0099	0.0055	17.5265	6.1715	0.741789	−0.31108	−0.21531
280	0.0093	0.00545	17.4335	6.117	0.759224	−0.31713	−0.21815
290	0.0088	0.00535	17.3455	6.0635	0.775719	−0.3231	−0.22066
300	0.0083	0.0053	17.2625	6.0105	0.791353	−0.32899	−0.22289
310	0.0078	0.0053	17.1845	5.9575	0.806047	−0.33486	−0.22479
320	0.0074	0.0052	17.1105	5.9055	0.819888	−0.34067	−0.22641
330	0.00695	0.00515	17.041	5.854	0.832946	−0.3464	−0.2278
340	0.0066	0.00515	16.975	5.8025	0.845193	−0.3521	−0.22893
350	0.00625	0.0051	16.9125	5.7515	0.856736	−0.35778	−0.22983
360	0.0059	0.00505	16.8535	5.701	0.867578	−0.36341	−0.23053
370	0.00565	0.00505	16.797	5.6505	0.877788	−0.369	−0.23104
380	0.00535	0.00505	16.7435	5.6	0.887416	−0.3746	−0.23137
390	0.00515	0.005	16.692	5.55	0.896503	−0.38017	−0.23153
400	0.005	0.00125	16.642	5.5375	0.906603	−0.38393	−0.23269
410	0.0048	0.00125	16.594	5.525	0.918188	−0.38531	−0.23521
420	0.00475	0.00125	16.5465	5.5125	0.929524	−0.38669	−0.23763
430	0.00465	0.00125	16.5	5.5	0.940775	−0.38808	−0.24001
